# The Mental Aspect of Epidemics

**Published:** 1851-04-01

**Authors:** 


### Art. II.—THE MENTAL ASPECT OF EPIDEMICS.

The history of plagues, like that of the sacking of great cities by hostile armies, is a theme full of the most mournful images. The physical and moral evils of such terrible catastrophes are seldom considered. The order and regularity, the comfort and wealth, in which we repose, exclude them from our thoughts; nor is it until some shock of nature or of politics awakes us with a start, that we are made aware of the terrible vicissitudes to which we are exposed.

It would be difficult to recapitulate the opinions of the various authors who have treated of epidemics and contagious diseases. For what use would there be in trying to arrange a chaos of hypotheses, built for the most part on conjecture, superstition, or empiricism? What, in fact, have we learnt on this perplexing subject from the *quid divinum* of Hippocrates, down to the fungoid theory of modern cholera? Cardan, Mercurialis, Valesco of Tarentum, and others, impute a divine anger or malignant influence to the conjunction of certain planets, which they assign as the efficient cause of epidemic pestilences. Van-helmont, Paracelsus, and the old German school, pretend that the contagious and epidemic principle consists in salt, sulphur, alkali, or arsenic, floating in the air. Schenck, Wirdig, Misald, and *les curieux de la nature*, have collected a great number of observations on this point, which are as worthless as they are ingenious.

Baillou, Sydenham, Ramazzini, Huxham, Tissot, Grant, Zimmerman, Lepecq, Monro, and Pringle, are the authors who have given us the deepest insight and the soundest reflections on this interesting topic. Some modern writers, indeed, have fancied that they have found out the cause of epidemics in certain states of the air, vitiated by a heterogeneous though unknown matter, which chemistry and natural philosophy have in vain endeavoured to discover in the constituent elements of the atmosphere.

Webster entered on some researches, for the purpose of proving the coincidence of epidemics with particular phenomena in nature, such as comets, volcanoes, earthquakes, &c.; but with what real benefit to practical medicine, it is not easy to perceive,—at least, we have not gained a single aphorism in addition to our present catalogue of them.

Every region of the globe is at times the seat of epidemics,—continents, islands, and even the ocean itself. The Matlazahuel, a kind of diapedesis, or bloody sweat, prevails among the savage tribes who wander upon the flanks of the Cordilleras. The Siamese of the old world, and the inhabitants of Massachusetts in the new, both fall victims to the yellow fever. The insular natives of the Maldives in the eastern, and the settlers of moist Cayenne in the western hemisphere, as well as our convicts at Botany Bay, in what is miscalled the *fifth quarter* of the world, are all of them liable to be carried off by fevers of a malignant type. In short, the snowy deserts of Siberia, the temperate and salubrious climate of Switzerland, the warm and humid levels inundated and fertilized by the Nile, the hot and dry provinces in the midst of Spain, the towering heights of Caucasus and the Alps, the immense plains of Poland, the shores of the Baltic and the Mediterranean, the marshy districts of the papal states, the beautiful and fertile champaigne lands of France and Lombardy, and the smiling vales of Tuscany,—all of them experience the baneful influence of epidemic maladies. Some, indeed, are indigenous, as the Siberian tara. But others perambulate the earth, such as the influenza and the cholera. The genius, epidemic agent, *ens epidemicum*, is a true Proteus, cloaking itself in every possible form of disease, and sparing not country, nor latitude, nor clime. The succession of the seasons, the various temperatures of the several zones, and the ever-changing winds of heaven, present no obstacle, offer no diversion, to its threatening and irresistible progress. We can watch, note down, and report the stations in its line of march; speculate on its deadly onset, and count the numbers of its slain;—nay more, we can look the portentous monster in the face, and touch its terrible existence with our hands: but respecting its intimate nature, its penetrating virus, we cannot pretend to anything better than a shrewd guess; and then, as to its specific treatment, we are forced to own that we know next to nothing.

No writings worthy of credit as scientific memorials have reached us, of a higher date than the close of the fifteenth century. Massaria, Arnaud de Villeneuve, Capivaccius, Gallus, Guy de Chauliac, Fracastor, and afterwards Zacutus Lusitanus, Ferri, and the Cardinal Gastaldi, have left us some interesting remains on the contagious maladies that happened in their time. Fracastor is the first who spoke of petechial fevers and epizootic diseases. Ramazzini, Lancisi, and Vallisnieri followed in his footsteps. The latter proposed (as Virgil had done before him) to slay the animals seized with contagious maladies on the spot, so as to intercept their propagation.

M. le Docteur Loudun, of Lyons, says, that the greater catarrhal diatheses of the present time are, in all probability, owing to the increase of cold and moisture observable in Europe ever since the great earthquake at Lisbon, as well as to the progressive debility of the human constitution, in consequence of the present mode of living, morals, habits, and the agitating events at the close of the last century and the beginning of this. And the late Dr. Prout considered, that, for the last thirty years, phlogistic or inflammatory states of the constitution have been decidedly on the decrease—a fact which accords with our own experience, extended over the same space of time. Dr. Beddoes had observed, as far back as 1807, the increase of pulmonary disorders, owing, as he remarked, to a state of atmosphere favourable to the development of asthenic diseases. Our private observations on this point certainly confirm us in the notion, that nervous debility has prevailed very extensively ever since the first appearance of the cholera in this country in 1832, while acute inflammatory affections have diminished in the same ratio. The object of this article, however, is not upon the *physical*, but the *moral* character of epidemics, with which, nevertheless, the foregoing remarks are not altogether irrelevant.

The passions of the soul have not unfrequently given rise to convulsive epidemics, or *dementia*, spreading by imitation. History, both ancient and modern, is full of examples. Pausanias makes mention of the maidens of Prætus and the women of Argos, who fancied themselves metamorphosed into cows. According to Plutarch, the girls of Miletus conceived a strong propensity for hanging themselves. M. Desloges, physician of St. Maurice in Valais, relates a similar epidemic, prevented from running to extremities by the timely exhortations of the *curé* of the place. Bonnet speaks of a transport of the same kind among the females of Lyons, inciting them to drown themselves. We have seen similar instances in our metropolis. Epidemics of persons *possessed* were common in Germany and France in the fifteenth and sixteenth centuries. That of the *Nonains* was notorious in Saxony, Brandenburg, and sober Holland. In the seventeenth century, the demoniacs of the rural district of Labour in Gascony, and the *possessed* of Loudun, made a great noise in France. Then came the convulsionists of Cevennes; and, in the last century, the fanatics at the miraculous tomb of the so-called *blessed* Paris; and, lastly, the crucifying women (*crucifiemens des femmes*) of Fareins in Dombes, 1786-88. These epidemic ecstasies were bad enough in their way, but nothing in comparison of those to which we will briefly refer.

The disaster of a large community infested by the well-known plague, of which Defoe has given so vivid a description when it happened in London, cannot be better understood than by calling to mind the manner in which it operated on the mixed population of Marseilles, about 100 years ago. What must have been the feelings of an entire population, driven to desert their dwellings, bivouac in the open country, or seek refuge on board the vessels anchored in the harbour! The magistracy and the religious orders abandoned the town, and none were left behind except the lowest class, the tradespeople, and the secular clergy, who, animated by the example of their excellent bishop, M. de Belzunces, continued to discharge their incessant and dangerous duties with the most heroic fortitude. A plan was formed for victualling the place, and the paupers and vagabonds were constrained to assist in burying the dead. Business of every kind was at a stand-still; disorders of all sorts were perpetrated, but most especially those of plunder and debauchery. It was a fearful scene; the streets and squares were obstructed with the dead and the dying, dragged from their doors, or thrown out of their windows, and left to expire or putrefy in the public way. A thousand persons died daily. The dogs prowled about the thoroughfares, and gnawed the naked carcases.

But what renders a plague peculiarly terrible, is the mental illusion that bewilders the populace in these critical moments. Alienation of mind forms, in the greater number of instances, the prominent feature of a genuine epidemic. Indeed, a plague of any kind is a species of madness. There is nothing too absurd, too cruel, too enormous not to be believed, and not only believed, but acted upon with the most terrific promptitude. The religious instinct, intensely excited, is the origin of all the extravagant and mischievous ideas usually broached in these appalling hours. Tumult is the result. And what can be done against a multitude maddened to frenzy on a single point, the most absurd and ungrounded in the world?—a fatuity too silly not to be despised, and yet too potent not to be shunned or destroyed, like a noxious reptile? Unhappily, such derelictions of reason constitute an ominous item in the accounts of epidemics; and the tale of many a stately city, ravaged by the plague, warns us to beware that, whenever the angel of death unfurls his black banner above our heads, superstition will not be slow to erect her hideous crest in front, while crime of the blackest die will dodge our heels at every step, and cowardice and despair beset us at every turn.

*Obstupuêre mentes et obduruerunt*, says Otho d'Arezzo. The ties of blood and friendship are broken—our animal nature reigns supreme. Some die drunk—others starving—and others, again, drop amid thoughtless pleasures. Public instruction is at an end,—Christianity itself is defunct,—its ministers die, so do the rulers, and the men of might. Husbands, having become widowers, seek to enter holy orders, for the sake of doing penance, or, perchance, from the sordid motive of possessing themselves of the unbequeathed wealth of the deceased clergy. The laws can no longer be put in force, for the courts of justice are vacant, and the culprits either escape the legal penalties due to their misdemeanours, or else they affect to punish themselves according to their own fancies. Remorse, that spiritual element uniting the future with the past, degenerates into a morbid desire of self-discipline, as soon as ever it is abandoned to its own mad caprices, and left uncontrolled by the rule of faith and the guidance of authority and discretion. Such are some of the dismal particulars drawn from ancient chronicles. Hence came the fanaticism of the middle ages,—the flagellants and others; and the rage which seized the world, when the Jews were suspected of having poisoned the air, and were consequently hunted to death and slaughtered in every part of Europe. At Essling, a whole congregation of them were burnt together in their synagogue. Mothers threw their infants into the flames, and then jumped in after them, to avoid a violent baptism at the hands of their infuriated persecutors. In vain did Pope Clement V. issue, from Avignon, a charitable brief in their favour: or the Emperor Charles IV. offer them a refuge within his dominions; or Albert, duke of Austria, threaten and inflict temporal vengeance on those implicated in these revolting crimes. Legislation was useless: the political mania was an epidemic beyond the reach of the triple mitre, the crown, or the sword.

But the biography of the human species is a burlesque as well as a tragedy. There was a profane song the children used to sing about the streets of Paris in the reigns of Charles VI. and VII., the words of which were these;—" *Votre . . . . à la toux, commère; votre . . . . à la toux, la toux!*" The tune, or the words, (those of a vulgar ditty, of course,) were imagined to be the cause of an epidemic catarrh, which threw almost every one prostrate on their beds. The symptoms were those of exhaustion and fever, which lasted about three weeks. They called it *La tac*, or *Horion*. Those who had recovered from it congratulated each other, jokingly, "*Par ma foi, tu as chanté votre . . . . à la toux, commère!*"—the word omitted in the text being an improper one. Nevertheless, high mass was *sung* daily in spite of the hoarseness and the cough. Men and women, especially those who were mothers, suffered severely, and were a long time in regaining their health and strength. There was no accounting for the disease, neither did the physicians seem to understand it. Superstition alone ventured to cut the knot in two, by confidently affirming that it was a curse upon those who had sung the profane song; and so prevalent was this gross idea, that if any one called on a friend just fallen sick with it, the usual exclamation was:—"*En as tu? Ah, par ma foi, tu as chanté la chanson!*"

With a scene of this flippant description, more like a vaudeville than a sick chamber, we may contrast one of the most terrible castral epidemics ever recorded by military surgery. It happened during the retreat of the French army from Moscow; and it will be easily understood how much any malady would be aggravated in the midst of such a disastrous combination of circumstances.

Wilna, over-crowded with troops hastily thrown into it, and at the same moment attacked by the Russians, became a scene of the most horrid carnage ever witnessed for several days in succession. The streets were choked up with the slain, and the French prisoners were thrust into the hospitals and the church of St. Casimir pell-mell. Notwithstanding the intense frost (28° Reaumur), typhus broke out among them, generated by the pestilential atmosphere arising from the putrefying corpses of the soldiery that had died from wounds, cold, hunger, and fatigue, in the midst of the most disgusting and unavoidable filth. The disease announced itself by headache, delirium, stupor, and anguish of the limbs, generally frost-bitten. In their delirium, the forlorn wretches beheld the cossacks charging down upon them in fury,—villages and towns glared with flames,—the frightful passage of the Beresina yawned before them,—and all the distresses of the retreat were repeated minutely before their inflamed and bewildered fancies. They imagined themselves preternaturally divided into a multitude of individuals, or surrounded with dreadful fiends to whom they were fastened, but from whom there was no escape, and by whom they were mocked and tormented with the most officious and loathsome assiduity. To their delirium was added a fiery heat and a burning thirst,—fury or apathy,—bloody spots, ulcers, mortifications;—such were the unparalleled horrors of this hell upon earth.

The Jews, who purchased or pillaged the chattels of the dead, paid dearly for their avarice by the penalty of disease and death. Entire families, of the first respectability, fell a sacrifice to the hospitality with which they treated the invaders of their country. Of 30,000 military, attacked by the malady, 25,000 died; and out of a population of 30,000 Jews, 8000 perished. In the hospitals, the confusion was beyond redress. The wisest and most humane measures were commanded by the Emperor Alexander; but in vain. The devotion and skill both of the Russian and French physicians were heroic to the last degree; but it was hopeless. Nor was it until death had very considerably reduced their numbers (40,000 persons, civil and military, were carried off by it), that proper remedies, food, nursing, &c. could be brought effectually into action for the assistance of the survivors. It was a time never to be forgotten.

By what particular course of events, or great moral lessons of weal or woe, the mass of mankind have been materially improved, it were hard to tell. Nevertheless, when we compare the present with the past, and review the social condition of the world, not four thousand, but only four hundred years ago, we are compelled to acknowledge that we are, both individually and collectively, a superior order of beings to what our forefathers were, either in the middle ages when the helmet, the cowl, and feudalism were rife, or in the days of the Persian monarchy, when Xerxes drove his servile myriads across the Hellespont at the beck of task-masters, with a lash in one hand and a bowstring in the other. Philosophers as well as religionists, however, are not wanting who are convinced that the world deteriorates, in morals at least, if not in intelligence, the older it grows, and that the last periods of the protracted history of our race will be worse than the first. In the ancient Sagas, or poems of the Brahmins, this discouraging presentiment forms the burden of their song; and of the four thousand years that have succeeded to the earliest date of their primeval legends, the concluding two, of which *we* are now living in the last, shall be, according to their vague predictions, the consummation of infidelity, misfortune, wickedness, and woe.* We are told upon authority, which the boldest contro-

* This prediction or prophecy, most probably of antediluvian origin, from the line of Cain, is verified in the fate of the Hindoos themselves. Their present condition, under the dominion of the Anglo-Norman race, is despicable, degenerate, and corrupt, when compared with their earlier dynasties, of which the Vedas sings. Their independence, their wealth, their martial prowess, their architectural splendour, are now no more. The spiritual teaching of Vishnu, however idolatrous and false, was eclipsed by the rationalistic reformation of Budda; and the modern Hindoo is no longer a chivalrous believer in the creed of Brahma, but the helpless dupe of a pantheism and fatalism conjoined, as withering as it is incurable.

versialist or philosophic inquirer may not dare to gainsay, that the terrestrial world "waxeth old as doth a garment;" from which we may rationally infer, that the general face of nature, like the features of a person advanced in years, exhibits the traces of decrepitude and decay,—so that, if one, born in the postdiluvian period, when this globe had just emerged, damaged indeed, but still fresh and young from the recent deluge, were to rise from the dead and appear among us, he would exclaim with unaffected surprise, that the sky, the air, the water, and the land were evidently the worse for wear, and apparently verging towards their final ruin and dissolution. In regard to the physical structure of our frames, it is a popular opinion, that in magnitude men are smaller now than they were formerly, and that our size has diminished even during the comparatively brief interval of the Christian dispensation; but that previously, men not only lived longer than they do at present, but that they were actually of much larger growth than ourselves; in short, that "there were giants in those days." It is the concurrent notion of the moderns, however, who have applied themselves to the investigation of this curious question, that our anatomical structure was originally cast in the same mould, and fashioned in the same proportions, both relatively and absolutely, as it still continues to be demonstrated in our dissecting-rooms and museums, and that this permanent fixedness of construction would appear to be attested by such human remains as have been discovered from time to time. It must be owned, indeed, that such remains are few and scanty, and that they scarcely ever exceed the date of a very modern antiquity.

This question was agitated in the early periods of learning, and the prevailing notion then was, that the primitive inhabitants of the earth were of larger growth than the later. Thus, St. Augustine, in his work *De Civitate Dei, Lib.* xv., *c.* 9, says, that the incredulous refused to believe that the stature of men, before the flood, exceeded that of those in his day, A.D. 427. Frequently he continues, from ancient tombs, whose contents have been laid bare by floods, violence, or time, I myself have beheld gigantic bones purposely exhumed, or exposed to light by chance.

Pliny considered (he adds) that, as time went on, mankind diminished in the same degree—the procreative powers of nature becoming exhausted. And the complaints vented by Homer, of the degeneracy of men subsequent to the siege of Troy, may be regarded, not so much as a poetic licence as an historic hint of some value from so accurate an observer as the author of the "Iliad" evidently was. Virgil, likewise, in his striking passage of the field Pharsalia, mentions the *ossa grandia sepulchri,* not as a pleasing fiction, but as a matter of fact with which his readers were well acquainted.* At all events, such testimonies prove a train of thought with which the world was already familiar. It has been objected that the bones mentioned by St. Augustine were merely the fossil remains of animals now extinct; but St. Augustine expressly says, *sepulchra convincunt;* nor was it likely that so keen, practised, and experienced an intellect, as that of the Bishop of Hippo's, could have been deceived in this particular. Moreover, let any one dispassionately examine the monumental remains lately collected and brought to Europe from Nineveh by Mr. Layard, and he must be convinced that they represent a race of men who, in their animal formation and propensities, were manifestly far superior to the average character of men in the present state of society.

Be this as it may, we cannot but conclude that, however much our bodies may have degenerated in lapse of time, our minds have, on the contrary, become more enlightened by the experience of ages. The manner in which the epidemic that visited us in 1849 was met and submitted to, is a decisive proof of this, and shows that the mental capacity and docility of the masses of mankind are very materially exalted in the scale of moral beings. Our Transatlantic brethren, the Americans of the United States, surpassed us, we must own, in this respect. No superstitious propensities were evinced on either side of the ocean—no fatal delusions, instigating the populace to public outbreaks of a terrifying nature—no disabling panics—no shameless libertinism,—nay, no profane outcry, or brutish infidelity. But everything was conducted with the most perfect self-possession—soberly, humanely, and discreetly. The best means, suggested by the best reason and knowledge, as far as they went, were listened to, adopted, and resolutely put into practice; while the heartfelt piety of the people was exhibited by the whole nation willingly relinquishing their temporal

* In one of those delightful passages with which the author of the Decline and Fall occasionally treats his reader, there is an allusion similar to that of Virgil's quoted above:—"From the first hour of the memorable twenty-ninth of May, disorder and rapine prevailed in Constantinople, till the eighth hour of the same day, when the sultan himself passed in triumph through the gate of St. Romanus. He was attended by his viziers, bashaws, and guards, each of whom (says a Byzantine historian) was robust as Hercules, dextrous as Apollo, and *equal in battle to any ten* of the race of ordinary mortals."—On the same day, the *muezzin* proclaimed the name of God and the prophet from the most lofty turret; and the altar, before which the last of the Cæsars had so lately bent in prayer, was now the high place of a Turkish mosque. As the sultan wandered through the desolate mansion of the great Constantine, he muttered a distich of Persian poetry: "The spider has wove his web in the imperial palace; and the owl hath sung her watch song in the towers of Afrasiab." The sacking of Constantinople was the closing scene in the downfall of the Roman empire—the most appalling and stupendous event recorded in history. Greek literature was diffused throughout Europe; Laura and Petrarch appeared upon the stage; the Portuguese opened a way to "the wealth of Ormus and of Ind;" and the asceticism of the middle ages disappeared, perhaps for ever.

concerns, and calmly offering up their petitions to Almighty God, on a solemn day, unanimously appointed by themselves for that purpose, with prayer and fasting. It is an historical fact: and, with the exception of the Ninevites, who repented in sack-cloth and ashes at the preaching of Jonah, we are not aware of any other instance on record in which a mighty empire demeaned itself in the same sublime, dignified, patient, and heroic attitude of attrition.

On a former occasion, under somewhat similar circumstances, more than two thousand years ago, when the plague depopulated Athens, fine examples of filial piety and generous friendship were at first displayed; but as the consequences were almost always fatal to the children and friends, they were but rarely repeated afterwards. Then the fondest ties were broken; the eyes about to close for ever, beheld on all sides nothing but the most profound solitude, and death no longer produced even a tear. This callous insensibility gave rise to unbridled licentiousness; the most splendid fortunes were left a prey to inexperienced relatives, strangers, or the populace; and the survivors, imagining they had but a short time to live, felt themselves justified in passing their remaining moments in the midst of pleasure.

An ungovernable imprudence of this description stigmatizes most of the fatal epidemics related in the histories of nations. A tone of mind, sufficiently composed and energetic to meet the emergency without alarm, is the only one that affords a chance of success in combating the malady; but it implies a mental cultivation of a very high and venerable order—such, indeed, as has rarely been met with, except in the later epochs of the Christian era. A remarkable instance of this sort presented itself at Nola, in the kingdom of Naples, 1815.

Upon the first intelligence of its having broken out among them, measures were instantly adopted for its extinction. The city was surrounded by two ditches, six feet wide and deep: the first was sixty feet beyond the houses, and the second thirty feet beyond the first. A body of troops guarded the entrances, the sentries being posted within sight of each other, and lighted at night by fires. There was a drawbridge to each entrance. It was death to pass, or communicate with, the guards. A hospital was established within the city, with a proper staff of officers, nurses, &c., habited in *toile cirée,* a mask, gloves, and wooden sandals. They used iron pincers for handing everything to the sick, and carefully abstained from contact. They oiled their hands, lived well, drank wine, and attended to their digestion. The foul linen, removed by pincers, was immersed in acidulated water, and offensive matters were instantly removed or burnt. Nitrous fumigations were used in the morning, and every evening the floors were sprinkled with an antiseptic fluid. The corpses were never touched except with the pincers, and were buried in quicklime without delay. Suspected parties were separated in a hospital of observation, from whence, if they showed infection, they were removed to the hospital-in-chief. Infected localities were surrounded with a barrier, and their respectable residents held in rigid quarantine. Parties of pleasure were interdicted; the churches closed; hotels, taverns, &c., shut up, under the severest penalties. Domesticated animals were destroyed; and the butchers were not permitted to slaughter their cattle within the city, nor to bring in their hides and offal. Contagious substances were burnt; the concealment of them being a crime punishable by martial law. The entire city was divided into sections, placed under committees; and all the inhabitants had to present themselves at their windows twice daily, and to quote and deliver up their sick. The committees acted as commissariats. The local reports, returned to head-quarters, were submitted to the closest scrutiny and deliberation. In six months the town was clear of disease; but a triple quarantine was persisted in for three months longer. 950 persons were attacked, 728 died.

By a diligent investigation of the circumstances of the case, an epidemic, however malignant, could scarcely withstand the sanatory measures of the present age. It requires the co-operation of the civil, military, and medical authorities, united in a police force, and acting together with firmness, severity, and agreement; without which, their allied efforts will be useless. The separation of the infected, the appointment of proper hospitals, the burning of the dead, the destruction of contagious articles by fire, daily fumigations and "swabbings" (to use a nautical phrase), killing the domestic animals—*e.g.*, dogs, cats, birds, &c.—inquisitorial visits, and an extensive commissariat, are the chief points to be carried into execution by a sanitary police. And the more vigorously such measures are enforced at first, the shorter will be the time of their disagreeable continuance.

Such a systematic plan for arresting the progress of disease does not appear to have been thought of by the ancients. Hippocrates is said to have stayed a plague at Athens and at Phocis, but we have no particulars of it, and most critics regard it as apocryphal. Judging from his "Epidemics," we may conclude that he knew but little about the matter. He excels in his prognostics and descriptions, but his practice is generally puerile, and sometimes ridiculous.

Before we part from our reader, let us ascend this flight of steps, and from the lofty battlements of history look down upon the deep-worn channel of ages. In the reign of Tarquin II. a plague raged at Rome, which made him send his two sons to Delphi.

During the contest respecting the fixed laws proposed by Arsa, the city was alarmed by violent earthquakes and fiery exhalations in the air. These natural phenomena were regarded as the forerunners of calamity. Superstition fanned the fears of the populace. A thousand optical delusions were imagined or seen, and supernatural voices fancied or heard in the night. Livy and Dionysius report that it rained raw flesh, and that birds of prey caught it piecemeal while falling, like snow,—probably aërolites or meteors. No particular plague broke out in consequence of these presages; but the state of the public mind was itself the plague.

Upon the reduction of Veii, the victory which procured such glorious popularity to the plebeian military tribunes, a pestilence broke out in the depth of winter. Its rise may be traced to the troops, who had suffered severely from the cold during the operations of the siege. On their return in triumph, the weather suddenly changed from extreme cold to oppressive heat. Dissipation generally characterizes a victorious army; malaria was rife; and the mortality was great among men and cattle. The effect on men's minds was anything but that of horror and despair, the disease caught them in a moment of success and exultation. The sybilline books being consulted, the duumvirs discovered an expiation as novel as it was harmless and cheerful, the Lectisternium. The statues of Apollo, Latona, Diana, Hercules, Mercury, and Neptune were taken down from their niches, and laid on three beds placed about a table, on which magnificent repasts were served up to those deities for eight days together. Private families imitated these public ceremonies. Every one kept open house for friends, strangers, and even enemies. Actions at law, animosities, &c. were suspended; prisoners were released from their chains and forced to join in the entertainments. Rejoicing was the order of the day; nor were the prisons opened again until the festival was over.

A pestilence arose (364 B.C.) without any apparent cause. The seasons were regular. The winter had not been too dry, neither had heat succeeded to cold unexpectedly. The summer was not damp, nor the vegetation sickly, nor had the Calabrian winds prevailed. All ranks were swept away—Camillus among the rest. It is probable that some meteoric agency was at work; for, two years afterwards, that chasm opened in the midst of the Forum, into which the young Curtius leaped. The gulf closed again; but the people threw loads of earth and rubbish into it, along with the corn, fruit, and other oblations for the gods. Fifty years after, during a triumphal procession, on account of the defeat of the Samnites, the city was again visited by a plague. The triumph of Fabius was interrupted by funerals, and the plaudits of the populace were mingled with lamentations for the dying or dead. Prodigies were in abundance—there were evil prognostics of all sorts—in short, a panic. Blood, honey, and milk had been seen to flow from the altar of Jupiter; and one phenomenon is recorded not unlike that of recent date—namely, that it rained *earth*, in the same manner as it was said to have rained *soot* in some part of Ireland, during the Asiatic cholera, in 1849. This black rain still remains to be accounted for. Was it the result of volcanic eruption? for after the eruption of a volcano in Nicarigua, 1835, ashes fell and darkened the day in Jamaica, 600 miles distant, and the same is related of Byzantium from the first eruption of Vesuvius.* Exactly thirty years later, another most dreadful plague fell upon the mistress of the world. The sybilline books implied that it was the result of some secret crimes. The suspicions of the crowd were awakened, and a vestal would have been sacrificed to their fury, had she not anticipated her fate by putting an end to her own existence. This popular outbreak reminds us of a similar one at Moscow, on the first appearance of cholera in that city, when the mob broke open the hospitals, killed or wounded some of the medical officers, and were at last dispersed by an armed force.

During the reign of the Emperor Maximin, the empire was afflicted with every evil—a drought, a famine, a carbunculous plague, followed by blindness. Bread was excessively dear—the deaths beyond number. The rich pawned their possessions for food, and were at last reduced to nothing. Ladies of quality begged from door to door, in splendid apparel, but with downcast looks. Others tottered along like spectres, till they dropped by the wayside, crying out for food. Those who had wherewithal to give, refused to do so, fearing lest they themselves should be reduced to a like necessity. The public ways were strewed with corpses, which the dogs preyed upon; and the survivors killed the dogs, lest the infuriated animals should turn upon the living and attack them. The pest directed its ravages chiefly among the higher classes. Whole families perished at once and were buried together; and the pagans declared that the only charitable persons were the Christians.

In another plague, fifty years later, in Nicomedia, there was an earthquake—buildings were overthrown, and a conflagration arose from their ruins. The bishops were assembled in council, at the same time.

* St. Augustine, *De Civ. Dei*, lib. iii. c. 31, mentions the rain of earth or chalk,—true stones, not hail,—as a phenomenon well-known to his readers. From an eruption of Etna, he says, the waters of the Mediterranean became so warm as to melt the pitch on the bottoms of ships. In Sicily, such a quantity of ashes fell, that the houses were covered with them, and broke down under their weight. This was at Catana. There was also a flight of locusts from Africa, which fell into the sea, and were washed up in such quantities on the shore that a pestilence arose from them in the kingdom of Masinissa. He says, that at Utica, out of 30,000 soldiers, only ten survived, c. 23. He mentions an epedemic madness among dogs, horses, asses, and oxen. The City of God is generally disregarded as nothing more than an elaborate religious disquisition; but it is, in truth, the best comment on Roman history extant. There is a French translation by Moreau, printed, Paris, 1845; but the Latin is pleasanter than the French.—Latin edit. tom. ii., Cologne, 1851.

A Persian ascetic, to whom miraculous powers were ascribed, and who had once been master of the lions under the Emperor, learnt by revelation or report, that the city, which he seldom condescended to visit, was being devastated beneath him by the plague, for he lived alone in a lofty turret in the citadel of Nicomedia. After one of the shocks of the earthquake, he was found dead in an attitude of prayer; and the plague ceased.

Palestine was convulsed by an earthquake, 419 A.D. A cloud rested on the Mount of Olives, and a supernatural figure, real or imaginary, appeared in it. The pagans beheld their clothes covered with glittering crosses, or what they mistook for such. The year before, there had been an eclipse of the sun, and the stars were visible at noon-day, followed by a drought, sickness, and a mortality among men and cattle. A luminous meteor was visible in the sky throughout the summer, and a strange fire fell upon earth, and was swept into the ocean by a hurricane. There was a panic, and the end of the world was supposed to be at hand. St. Augustine wrote to prove that the alarm was needless.

The most formidable plague on record is that which occurred 542-594 A.D. Such was the universal corruption of the air, that the pestilence which burst forth in the fifteenth year of Justinian was not checked or alleviated by any difference of the seasons. In time its first malignity was abated and dispersed; the disease alternately languished and revived; but it was not till the end of a calamitous period of fifty-two years that mankind recovered their health, or the air resumed its pure and salubrious quality. No facts have been preserved to sustain an account, or even a conjecture, of the numbers that perished in this extraordinary mortality. During three months, five, and at length ten, thousand persons died each day at Constantinople; many cities of the east were left vacant; and in several districts of Italy the harvest and the vintage perished on the ground. The triple scourge of war, pestilence, and famine, afflicted the subjects of Justinian, and his reign is disgraced by a visible decrease of the human species, which has never been repaired in some of the fairest countries of the globe.

But as an instance of popular hallucination, that which took place at Milan would surpass belief, except from the acknowledged testimony on which it rests. The entire population was afflicted with second sight, or phantasmagoria. On a calm day, when the western front of the duomo reposed against the blue sky of a Lombard summer, a terror-stricken citizen saw, in the middle of the square, a carriage drawn by six horses: within, was a person of majestic mien, dark complexion, eyes inflamed, and lips compressed and threatening. The spectator entered the carriage: it drew up before the gates of a magnificent palace.

He alighted, and, entering its spacious halls, beheld a strange scene of horror and delight. Pale ghosts sat in council: he was tempted by a vast bribe of gold to accept a small vase of poison, for the deadly purpose of employing it against his fellow-citizens. He refused; the scene vanished; and he found himself once more in the empty square, before the duomo, beneath the deep blue sky, the same as ever. This tale was repeated by a hundred lips. The carriage, the palace, the phantom council, were listened to and believed. Milan was tossed in the stupor of an uneasy dream. The pestilence was explained. The dust on the pavement was a poisonous powder. But who were the poisoners? They found an old man dusting a bench—was it he? They fell upon three French artists admiring the venerated duomo, and touching it with their hands,—surely it was they! Such was the distempered fancy that peopled the ancient streets of Milan. Frederic Borromeo was appealed to, and he replied that they were the dupes of a panic.

Mortality, in its ordinary form, is regarded with indifference by the crowd. Man passeth to his long home, but no one layeth it to heart; but in the gloom of a general calamity, the consciousness of an impending evil, from which there is no escape, becomes, when deepened by despair, a stupefying element, that benumbs the faculty of reason, unnerves the senses, and deranges the order of the understanding. The foregoing accounts, which have been copied, almost verbatim, from Fleury, Gibbon, Hooke, Manzoni, and Ozanam in his history of epidemics, are, strange indeed, and melo-dramatic as they may appear, entirely illustrative of the mania, partial or complete, usually attendant on the progress of a pestilence.

The old astrologers might be correct in their mystical surmises, although mistaken in the inferences they ventured to draw from them. Their prognostications may have been hazarded upon data, fallacious because they were partial, not because they were absolutely unfounded. There is no question that our lives depend on telluric, meteoric, and astral influences, to a degree rejected by the dull philosophy of the last age, and received with attention and scientific exactness, but tardily, by the present. It is impossible to exclude sidereal phenomena from playing a chief part among the operations of what may be strictly denominated "*vital dynamics*." Indeed, the shock imparted to the mind, both individually and collectively, by the mere occurrence of strange appearances out of the course of nature, is no trifling ingredient to help us in accounting for the political, moral, and sanitary condition of the world; evolving, as it does, in bold and decided attitudes, inherent energies of the soul, which would, like the hidden elements of the terrestrial globe, have remained, under ordinary circumstances, latent, invisible, and unknown. For a comet may, as the poet says, "shake from its horrid hair both pestilence and war," by altering the electro-chemical affinities and barometrical pressure of the atmosphere, and thus excite the sensorial and sanguineous functions of our frames to the last degree of national frenzy. A solar eclipse refrigerates that portion of the earth's surface over which the lunar shadow passes; earthquakes disturb, if they do nothing worse than disturb, the accustomed direction and velocity of winds and currents of air; and volcanoes emit mephitic vapours, and dust, and sheets of flame, poisoning at once and overheating vast districts of inhabited countries, in a manner as inimical to life as it is subversive of that diurnal and regular routine of health, so essential to animal and vegetable organizations. The mind corresponds to the vigour or debility of the body,—excitement, superstition, and fear, are the invariable coincidents or consequences of particular ailments, or physiological conditions. Multitudes may be staggered by an apparition or visual change in the aspect of things, and their blood be curdled or inflamed by the invasion of an epidemical panic. All classes are merged in the common evil. Princes and rulers sympathize with the crowd; and, instead of riding on the whirlwind and directing the storm, may be, like the meanest of their subjects, hurried away by the vulgar impulse, and lashed on instinctively to the strange, unnatural, incontrollable issue of events. Viewed in this light, the history of mankind is, in its final causes, nothing more than the natural history of the universe, of which man constitutes an integral part equally with every other organic and inorganic substance, and submits to a destiny in accordance with the revolutions of planets (of which this earth is one) around the sun, and in compliance with appointed changes, proceeding with a giant's stride among stars and systems of stars, infinitely remote in the boundless regions of space—where comets wander with amazing and perplexing precision, and constellations appear and disappear in a mode that baffles the wits of the most refined philosophy. That man made of clay (*memento, homo, quia pulvis es, et in pulverem reverteris*) with a soul full of celestial aspirations, should, for the short space of three score years and ten, be doomed to a lot little above that of an earth-worm, is an enigma only to those who have not studied the discoveries of science in their moral relations, nor turned to behold, with the eye of faith, "the works of the Lord, and the wonders that He doeth in the deep!"

